# Draft Genome Sequence of Paenibacillus odorifer V, Isolated from the Fecal Material of a Rabbit

**DOI:** 10.1128/mra.00405-22

**Published:** 2022-06-21

**Authors:** Veronica Perez, Zina R. Haywood, Richard W. McLaughlin

**Affiliations:** a General Studies Department, Gateway Technical College, Kenosha, Wisconsin, USA; University of Rochester School of Medicine and Dentistry

## Abstract

Here, we report the draft genome sequence of Paenibacillus odorifer strain V, which was isolated from the fecal material of a rabbit living in the wild. The genome size is 6,863,583 bp, with 44.35 mol% G+C content.

## ANNOUNCEMENT

Paenibacillus odorifer is a Gram-positive, motile rod which is capable of producing oval-shaped endospores. The bacterium has been isolated from a variety of sources, such as wheat roots, leeks, and courgettes (1). In a recent study, *P. odorifer* was the most common *Paenibacillus* species, among a group of over 1,200 isolates which were collected from milk and other dairy environments (2). In this study, *P. odorifer* strain V was isolated from the fecal material of a rabbit living in the wild using the ethanol shock method (3). Bacteria were plated onto tryptic soy agar (TSA) (Hardy Diagnostics) and incubated at 30°C for 48 h under both aerobic and anaerobic conditions.

For genomic sequencing, a colony of *P. odorifer*, which was grown aerobically at 30°C for 48 h on TSA, was sent to the Microbial Genome Sequencing Center (MiGS; https://www.migscenter.com/). DNA was isolated using a ZymoBIOMICS DNA kit (Zymo Research). Sample libraries were prepared using the Illumina DNA prep kit and protocol, with IDT for Illumina DNA unique dual indices (UDI), and sequenced on an Illumina NextSeq 2000 instrument, producing 2 × 151-bp reads. Demultiplexing, quality control, and adapter trimming were performed using BCL Convert v3.9.3 (4). The genome was assembled using SPAdes v3.15.4 (5) and annotated using the RAST toolkit (RASTtk) release 2018-0531 (6). Default parameters were used for all software.

In total, 73 contigs were constructed, totaling 6,863,583 bp, with an average G+C content of 44.35%. The *N*_50_ length is 719,313 bp, and the coverage is 100×. The genome contains 6,273 protein coding sequences (CDS), 84 tRNA genes, and 6 rRNA genes.

To determine the identity of the bacterium, the average nucleotide identity (ANI) value for our isolate was determined by submitting the draft genome sequence to the ANI calculator (7). When strain V was compared to the reference strain *P. odorifer* DSM 15391, a value of 97.19% identity was obtained, which suggests that strain V belongs within the species *odorifer*. No plasmids were detected as determined using PlasmidFinder v2.0 (8) with default parameters. In addition, using the Pathosystems Resource Integration Center (PATRIC) pipeline, we identified open reading frames (ORFs) responsible for putative siderophore production and fimbria and hemolysin synthesis. This draft genome sequence could in future be compared to other genomes from both dairy and nondairy sources for a more complete understanding of the genome features of the bacterium.

### Data availability.

This whole-genome shotgun project has been deposited at DDBJ/ENA/GenBank under accession number JALLFV000000000. The version described in this paper is version JALLFV010000000. The raw reads were deposited under SRA accession number SRR18776294, BioProject accession number PRJNA824532, and BioSample accession number SAMN27407113.
